# Light in the Darkness: Responses to Light and Diel Activity Rhythm in an Eyeless Cave Flatworm (*Dendrocoelum italicum*)

**DOI:** 10.1002/ece3.71584

**Published:** 2025-06-18

**Authors:** Benedetta Barzaghi, Raoul Manenti, Gentile Francesco Ficetola, Roberta Pennati, Andrea Melotto

**Affiliations:** ^1^ Department of Environmental Science and Policy University of Milan Milano Italy; ^2^ Laboratory of Subterranean Biology “Enrico Pezzoli”, Regional Park of Monte Barro Galbiate Italy; ^3^ Department of Botany and Zoology Stellenbosch University DST‐NRF Centre of Excellence for Invasion Biology Matieland South Africa

## Abstract

Troglobiont species show common traits derived from adaptation to subterranean life. Due to lack of light in cave environments most troglobiont species show eye reduction or even loss, often accompanied by a disruption of diel rhythmicity. Although cave adaptation and eye absence generally imply the loss of capability to perceive light, several cave‐adapted species have retained this function, showing some degree of phototaxy or rhythmicity. Flatworms are ubiquitous in natural habitats and the response to light or diel rhythmicity of surface‐dwelling species has received considerable scientific attention in the past and is increasingly studied. Conversely, responses to light stimuli have been poorly investigated in eyeless troglobiont flatworms. Here we coupled field monitoring and behavioural experiments to investigate phototactic responses and variation in diel activity patterns in the troglobiont eyeless flatworm *Dendrocoelum italicum* . We tested 27 *D. italicum* individuals from ‘Bus del Budrio’ cave, measuring their response to light stimuli in a semi‐obscured experimental arena under different light‐exposure treatments: dim light, bright light and darkness. Besides, during a 2‐year period we conducted 18 visual‐count surveys to monitor daytime and nighttime flatworm abundance in the cave. Behavioural tests showed that flatworms selected the darker side of the arena under both bright and dim light exposure, revealing a marked negative phototaxis. Field surveys revealed that flatworm abundance was significantly affected by the diurnal cycle, with a higher number of individuals visible during the night. These outcomes offer interesting insights on adaptation to cave environments, showing that key functions, such as the capability to respond to light or diurnal cycles, can be retained in troglobiont species and call for future investigation assessing the potential adaptive roles of these traits in mediating their exploitation of subterranean environments or their interface with the surface. Besides, this study proposes an effective in‐field method for conducting studies on subterranean fauna responses to light stimuli.

## Graphical abstract

Loss of non‐functional and costly structures associated with surface conditions is favoured during adaptation to cave environment. We show that eyeless cave flatworms express photophobic responses to light stimuli and retain diel rhythmicity in activity patterns. Typical surface‐related functions can be retained by cave‐adapted species, suggesting for potential adaptive roles mediating the exploitation of underground environments.

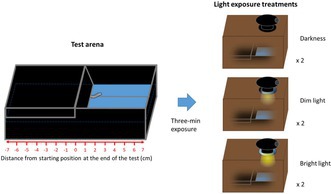

*It could work!*

(Young Frankenstein, 1974)

## 1 Introduction

Subterranean organisms, even when belonging to broadly different evolutionary lineages, tend to show parallel or convergent evolution for numerous physiological and morphological traits. Common adaptations include low metabolism, specialised sensory systems, depigmentation and loss of eyes (Romero 2009). During the process of adaptation to new ecological conditions, such as when colonising novel habitats, maintenance, regression or loss of traits originated under different selective pressures depend on the complex interactions among multiple factors shaping evolutionary trajectories (Jeffery 2010; Retaux and Casane 2013; Sumner‐Rooney 2018; Tierney et al. 2017). On the one hand, features typically related to surface habitats (e.g., pigmentation, visive structures, circadian rhythm) are supposed to disappear or regress in cave organisms due to patterns of neutral mutation and genetic drift (Wilkens 2010), direct selection against costly non‐functional structures (Moran et al. 2014, 2015) or indirect selection caused by adaptive shifts in other traits (pleiotropy) (Jeffery 2009, 2010; Yamamoto et al. 2009). On the other hand, maintenance of some adaptive value even in the subterranean environmental context (Fišer et al. 2016; Wang et al. 2023), reduced time since colonisation (Niemiller, Fitzpatrick, et al. 2013; Niemiller, Graening, et al. 2013; Wang et al. 2023) and indirect positive selection mediated by pleiotropic effects (Langille et al. 2019; Tierney et al. 2015) are among factors proposed to favour the retention of surface traits in cave habitats. Trait variation in cave‐adapted organisms is shaped by key selective pressures and peculiar physical features typically associated with the subterranean environment and groundwaters, such as the paucity of trophic resources, the microclimatic stability and the absence of light (Culver and Pipan 2009; Romero 2009, 2020). In particular, total darkness is a main driver of the selective filters acting on animals exploiting subterranean environments (Fišer et al. 2023). How troglobionts (terrestrial obligate subterranean‐dwelling organisms) and stygobionts (obligate groundwater‐dwelling organisms) react to light occurrence is one of the most popular topics that has been investigated since the dawn of cave biology research, with numerous experiments performed on a large variety of animals (Vandel 1964). Light can generate both attraction (positive phototropism/phototaxis) and avoidance in animals (negative phototropism/phototaxis). Positive phototaxis seems generally absent in obligate subterranean‐dwelling organisms, as attraction for light would result in a stimulus to leave the underground realm (Vandel 1964). This is not surprising as light exposure is detrimental to many depigmented cave‐adapted organisms, and damaging effects of light have been reported for different troglobiont species. For example, 15‐min exposure to sunlight can kill the cave‐dwelling beetles of the genus *Laemostenus* (Bernard 1932). Similarly, a two‐to three‐day exposure to constant light of 20,000 lx (typical ambient light intensity, with clear sky at midday) is lethal for depigmented amphipods of the genus *Niphargus*, whereas pigmented surface‐dwelling amphipods of the genus *Gammarus* can resist for 25 days under a constant light of 40,000 lx (Ginet 1960). Indeed, the negative phototaxis observed in most troglobiont/stygobiont species (Vandel 1964) has been proposed as an advantageous mechanism to prevent their dispersal into surface environments, where UV rays may be particularly detrimental due to the absence of pigmentation or where they can encounter other selective pressures they are poorly evolutionarily equipped to face (e.g., strong predation and competition, increased variability in environmental conditions) (Fišer et al. 2016; Galbiati et al. 2023; Vandel 1964; Wang et al. 2023). Nonetheless, surface habitats and their interface with caves can also offer important ecological opportunities for subterranean organisms, for instance in terms of richer trophic resources availability (Manenti and Piazza 2021; Manenti et al. 2024, 2025). Thus, in transitioning zones or areas close to the surface, light perception may play a key role in mediating the exploitation of these environments by troglophile and troglobiont species (Fišer et al. 2014; Friedrich et al. 2011; Hoenen and Marques 1998; Mammola and Isaia 2018; Manenti, Forlani, et al. 2023), for instance by favouring their access only in periods characterised by less risky or more suitable conditions (i.e., during night) (Manenti and Barzaghi 2021; Manenti, Forlani, et al. 2023). Although generally living in complete darkness, being able to respond to light stimuli may therefore be important for subterranean organisms and possibly retain some adaptive function, for instance in terms of microhabitat selection.

Several examples of subterranean species responding to light stimuli exist, including vertebrates such as cave fishes (Berti and Messana 2010; Frøland Steindal et al. 2023; Thinès 1953; Vandel 1964), some stygobiont salamanders (Dubois 1890, 1892; Hawes 1945) and numerous invertebrates, especially arthropods, such as beetles (Friedrich et al. 2011), spiders (Wang et al. 2023), *Niphargus* amphipods (Borowsky 2011; Fišer et al. 2016; Galbiati et al. 2023), isopods (Banta 1910), crayfishes (Park et al. 1941) and collembola (Paclt 1956). However, there are also some cases of troglobiont/stygobiont species that do not react to light, remaining unaffected by luminous stimuli. Those cases include some cave salamanders (Vandel 1964), isopods (Horváth et al. 2021), few dytiscid beetles of the genus *Paroster* (Langille et al. 2019) and even a flatworm (Buchanan 1936). Besides, for a vast number of subterranean‐dwelling animals, tests on the existence of a phototaxis have never been performed (Friedrich 2013). Moreover, the existing studies rarely compare cave organism reactions to those of the closest surface‐dwelling relatives (Banta 1910; Fišer et al. 2016; Manenti, Galbiati, et al. 2023) nor differentiate between individuals belonging from deep underground areas and those living at the edge with the surface (Manenti and Piazza 2021). Even rarer are studies coupling laboratory tests with ecological field surveys or research linking capability to respond to light and the expression of other typical troglomorphic traits. Nonetheless, some interesting examples exist (Fišer et al. 2016; Gallo and Bichuette 2018; Tierney et al. 2015; Wang et al. 2023). For instance, Gallo and Bichuette (2018) show the occurrence of responses to light in both cave adapted and surface diplopod species, and the existence of a negative correlation between eye‐regression and response to the stimuli (Gallo and Bichuette 2018), whereas a review performed by (Friedrich 2013) on multiple cave taxa highlights the presence of a correlation between the regression of the visual system and the reduction of diel rhythmicity responsiveness.

The occurrence and modulation of circadian rhythms is strongly linked to the perception of light in surface animals (Beale et al. 2016); indeed, light is generally the main synchronising cue (‘zeitgeber’) of day‐night variation in organisms' biological clock (Vaze and Sharma 2013). In subterranean animals the absence of diel activity rhythms is often correlated with the absence of the visual system (Friedrich 2013; Gallo and Bichuette 2018; Pasquali and Sbordoni 2014), with evidence suggesting rhythmicity loss can provide cave‐adapted organisms important advantages in terms of metabolic energy saving (Moran et al. 2014). However, both the occurrence of light detection systems and behavioural patterns regulated by the biological clock in cave species can be conserved (Beale et al. 2016; Friedrich 2013; Pasquali and Sbordoni 2014), with activity variation between day‐light periods especially in individuals/populations exploiting the borders with the surface (Manenti and Barzaghi 2021). Indeed, the maintenance of diel rhythm activity may vary with time or degree of adaptation to cave life and may depend on retention of some functionality or derive from evolutionary constraints associated to the physiological and metabolic processes it affects (de Souza et al. 2024; Friedrich 2013); diel rhythmicity in cave organisms can be endogenously generated but also modulated by external cues, such as light or temperature when in proximity or contact to cave entrance, or other zeitgebers that might be available in these environments (e.g., rhythmic predator/prey activity or food availability) (Menna‐Barreto and Trajano 2014; Pasquali et al. 2007; Soriano‐Morales et al. 2013; Stringer and Meyer‐Rochow 1997).

Although generally neglected, cave flatworms are common stygobionts species (Barzaghi et al. 2021; Manenti et al. 2018; Romero 2009) that can be of particular interest to address evolutionary questions regarding adaptation to the cave environment and to investigate the potential adaptive significance of light perception in subterranean‐dwelling animals, the mechanisms allowing it and the implications on their diel activity rhythms (Protas and Jeffery 2012). Indeed, flatworms are easy‐manageable model animals that have been used in many studies to address a variety of biological inquiries, and their capacity to respond to light has long piqued scientific curiosity. In his laboratory at the Castle of Wray in Windermere, Ullyott (1936a, 1936b) demonstrated that flatworm orientation was mediated by light, showing that light avoidance persisted in eyed‐deprived flatworms, and, together with evidence from earlier experiments (Parker and Burnett 1900; Walter 1907), set key bases for the investigation of extraocular vision. However, while the epigean flatworms have been well studied the same cannot be said for the cave ones. In one of the few reports, Vandel (1964) states that cavernicolous flatworms are extremely sensitive to light and underlines that they quickly dissolve themselves when exposed to light, making particularly difficult their picturing and filming. To date, the existing information on cave flatworm responses to light remains generally scanty and mostly anecdotal. In particular, research on the behaviour of stygobiont flatworms or evaluating their response to light stimuli in the context of behavioural ecology is extremely limited, while, to the best of our knowledge, there is no study explicitly investigating diel activity rhythms and its relationship with responses to light in cave flatworms.

In this study, we integrated experimental assays with previously available field observations in natural conditions (Manenti et al. 2019) to test whether cave‐dwelling flatworms with typical troglomorphic traits (i.e., cave‐induced adaptations) are responsive to light stimuli and show variation in the pattern of diel activity compatible with circadian rhythmicity. To address these questions, we focused on *Dendrocoelum italicum*, a steno‐endemic cave flatworm, totally eyeless and depigmented, that inhabits groundwater in a localised karstic system of northern Italy (Manenti et al. 2018; Vialli 1937). First, we evaluated whether the number of active individuals in natural conditions varies between night‐ and daytime. Second, we used experimental tests to assess whether flatworms show behavioural responses to different levels of light intensity, and whether light influences their position within experimental arenas.

## 2 Methods

### 2.1 Study Area and Focal Species

**FIGURE 1:**
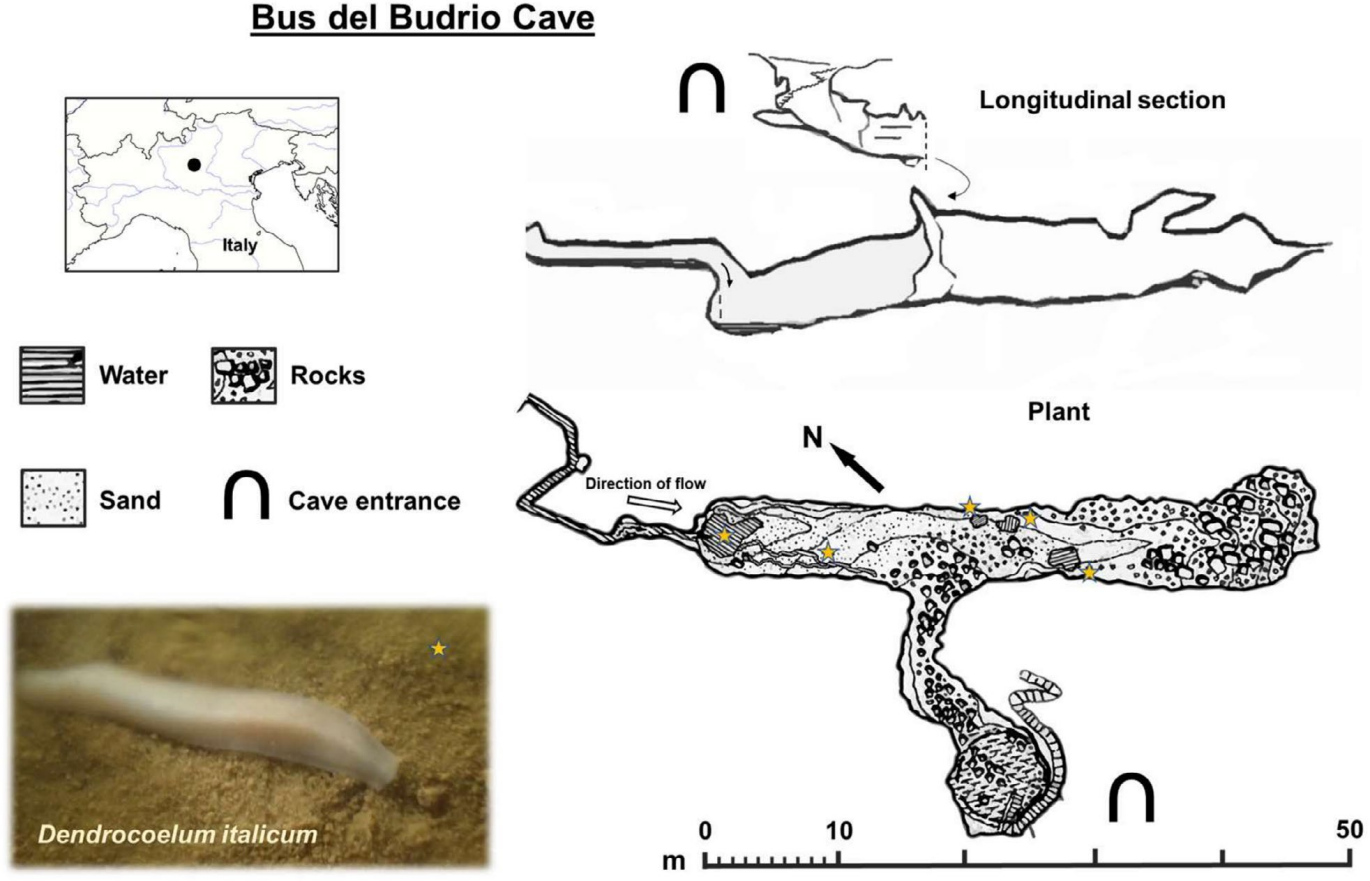
Study site. Plan and longitudinal section of the Bus del Budrio Cave, where we conducted both field surveys and behavioural tests. The water bodies used by flatworms and monitored to assess day/night abundance are indicated by orange stars. At the bottom left a picture shows adult individual of *Dendrocoelum italicum* (Bus del Budrio Cave, 2017).

The study was conducted in the regional natural monument called Upland of Cariadeghe in the South‐eastern Prealps (Lombardy, Italy). This karstic plateau hosts complex cave systems, with multiple interconnected underground waterbodies. Many natural openings occur in the area, providing numerous interface areas/contact zones between subterranean and surface habitats. Some of these caves were historically used for milk storage and cheese production by the local human populations. The plateau represents a hotspot of subterranean biodiversity as it harbours numerous troglophile and troglobiont species, including several steno‐endemic species, such as the cave‐dwelling flatworm *Dendrocoelum italicum* , which is the subject of this study. This cave flatworm was discovered in 1937 in the Bus del Budrio cave (Vialli 1937) (Figure 1) and seems to have an extremely narrow distribution. This species is only known for the study site, even though recent observations of putative *D. italicum* individuals in a nearby artificial gallery (~4 km distance from the Bus del Budrio cave, with no connection known) suggested that this species can be more widespread in the aquifers connecting the cave systems of the Cariadeghe's plateau (Manenti et al. 2018). In the Bus del Budrio cave, this species is the only cave flatworm present and inhabits the muddy substrate of subterranean watersheds, like narrow streams, puddles or small pools (Manenti et al. 2019), and shows classical troglomorphic traits (Vialli 1937), such as depigmentation and the absence of ocelli, the visive structures that are present in surface flatworms. The Bus del Budrio cave, where individuals were collected for this study, harbours a stable *D. italicum* population that has been increasingly monitored in recent years, and has also benefitted from habitat‐restoration interventions to ensure persistence of the main natural pool present in the site, which was heavily reduced in size by artificial water‐catching (Manenti et al. 2019). The cave consists of an elongated chamber (43 m long, 6.5 m height, average width ~4 m) connected to the surface by a small curving central passage (~5 m) ending in an external large and steep sinkhole, from which light filters in the immediate proximities of the cave entrance. Within the main chamber, a narrow stream together with several semi‐temporary and persistent pools intermingle in the muddy substrate of the cave and constitute the habitat of *D. italicum* . During day, light filters from the cave's opening reaching the inner end of the connecting passage, but watersheds where flatworms are found are essentially in darkness (maximum lux: 0.04, assessed with a PCE‐170 luxmeter).

### 2.2 Flatworm Abundance and Diurnal Cycle

Between February 2016 and January 2018, Manenti et al. (2019) assessed *D. italicum* abundance during 18 visual count surveys performed both during daytime and night. The count of visible individuals is an effective method to assess variation in species activity in the field (Aguzzi et al. 2013; Manenti, Galbiati, et al. 2023; Manenti et al. 2016; Myers et al. 2016). Moreover, *D. italicum* is an active predator that wanders on the substrate to search for prey (e.g., small crustaceans), which it traps through its mucus secretions. Thus, quantifying the number of visible individuals offers a good proxy to measure the level of activity in this species. Visual count surveys were conducted over a duration of 30 min and involved one or two operators who meticulously counted the flatworms while observing each pool from the external periphery, thereby minimising the disturbance, while preventing water turbidity and avoiding inadvertent contact with the flatworms themselves. Day counts were performed between 11 a.m. and 3.30 p.m., while night surveys were performed after dusk, between 9 p.m. and midnight. In total, 13 daytime surveys and 5 night surveys (see Table 1). The surveys performed by Manenti et al. (2019) aimed at testing the effectiveness of restoration activities performed in the study site and did not explicitly focus on the diel activities of *D. italicum* . Here we refined previous analyses on these data and discarded two surveys during which pool turbidity prevented accurate individual count.

**TABLE 1:** Results of field surveys.

ID survey	Date	Season	Day phase	N operators	Water condition	N flatworms
1	2016‐02‐04	Winter	Day	1	Clear	49
2	2016‐04‐22	Spring	Day	2	Clear	69
3	2016‐07‐27	Summer	Night	1	Clear	109
4	2016‐11‐15	Autumn	Day	2	Clear	41
5	2016‐11‐18	Autumn	Day	2	Clear	8
6	2016‐11‐27	Autumn	Day	2	Clear	43
7	2016‐12‐03	Autumn	Day	1	Clear	73
8	2016‐12‐04	Autumn	D ay	2	Turbid	6
9	2017‐01‐25	Winter	D ay	2	Turbid	7
10	2017‐03‐19	Spring	Day	2	Clear	52
11	2017‐03‐22	Spring	Night	1	Clear	109
12	2017‐03‐30	Spring	Day	2	Clear	26
13	2017‐03‐30	Spring	Night	2	Clear	58
14	2017‐03‐31	Spring	Day	2	Clear	43
15	2017‐03‐31	Spring	Night	2	Clear	91
16	2017‐04‐01	Winter	Day	2	Clear	31
17	2018‐01‐24	Winter	Day	2	Clear	65
18	2018‐01‐24	Winter	Night	2	Clear	81

*Note:* We report date, season, starting time of the survey and day phase (night or day), number of researchers involved in each survey, water visibility during surveys and the number of *D. italicum* individuals observed during visual counts. Two surveys (8 and 9, underlined text) were excluded from the analysis as turbid water conditions hampered accurate visual count. Surveys 1–7 were performed before a restoration intervention conducted to preserve the flatworm habitat, whereas surveys 8–18 occurred after it (see also Manenti et al. 2019).

### 2.3 Behavioural Response to Light

**FIGURE 2:**
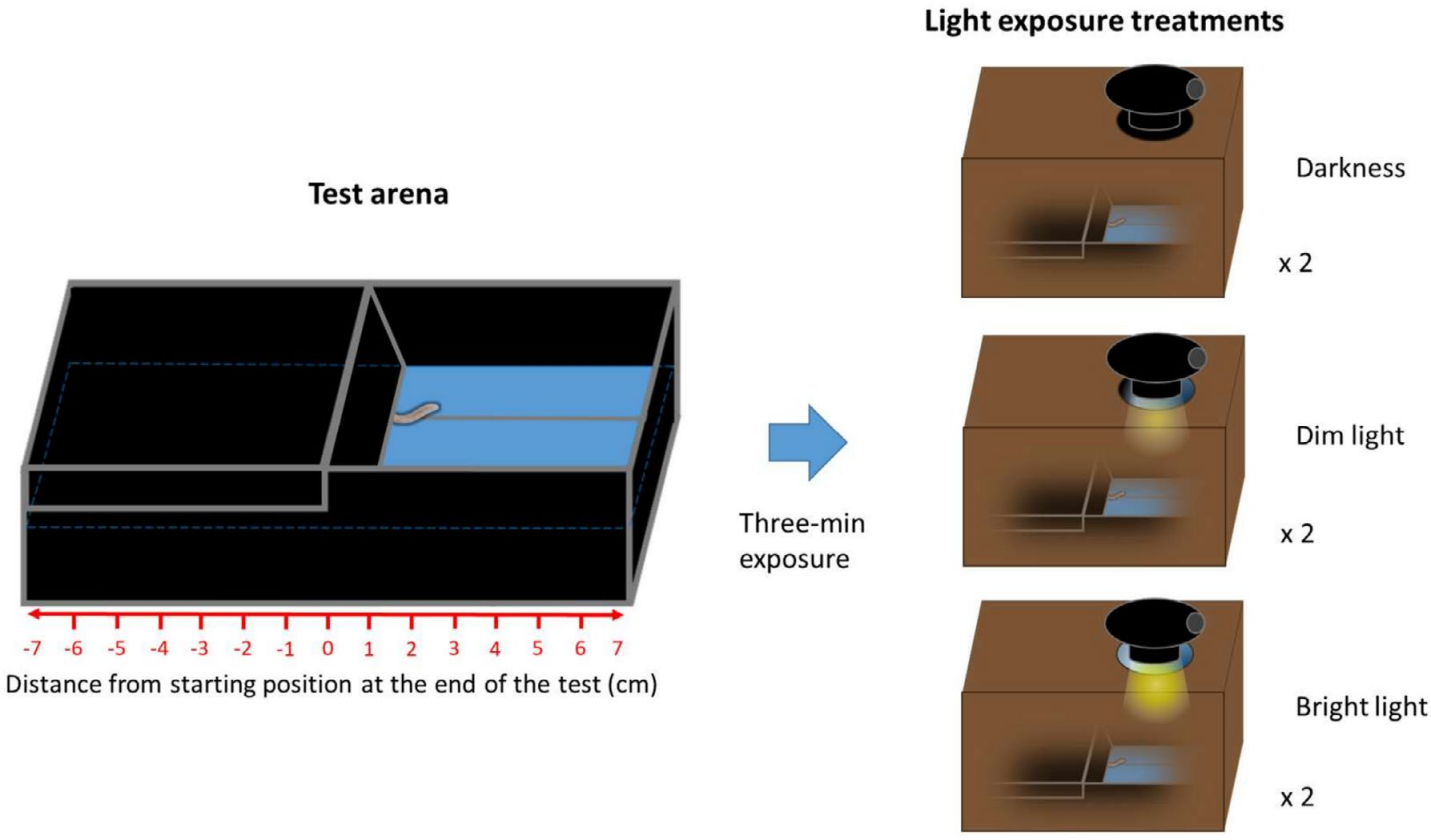
Setting of the behavioural test. Figure showing experimental arena (in black) and light‐exposure treatments to which *Dendrocoelum italicum* were exposed. The test arena was filled with 1.5‐cm of cave water and provided with a lid covering half of the arena until the water surface (dark side). A graduated scale on one side of the test arena allowed measuring the distance from the arena centre (starting position) reached by the flatworm at the end of the 3‐min test. Each flatworm was exposed twice to three treatments: total darkness, dim light and bright light. Light was provided with a lamp illuminating the light side of test arena through a hole performed on a coverage box (in brown) placed upon the test arena (in transparency in the figure).

The capability of *D. italicum* to respond to light stimuli was assessed performing behavioural tests on 27 adult individuals. As light intensity can affect behavioural responses of surface flatworms, typically increasing their photophobic reaction (i.e., light aversion) and travelling speed (Davidson et al. 2011), we compared the effect of the exposure to different light intensities, which were also intended to simulate potential conditions that could be experienced in different environments (surface, ecotone/twilight zone, cave) and during different day phases. Tests were conducted on a single day (between 3:00 p.m. and 9:40 p.m. of May 16 2019) inside the Bus del Budrio cave. This allowed preventing stress or damages to these fragile animals deriving from of transportation or acclimation to new artificial conditions. Behavioural tests were performed in complete darkness (≤ 0.01 lx illuminance) in a section of the cave located at least 10‐m far from the small passage connecting the inner chamber and the entrance. Behavioural tests consisted in measuring flatworm position in a obscured experimental arena under three different light‐exposure treatments: dim light, bright light and darkness (Figure 2). For light‐exposure treatments we used a 5 W Seoul led Luxen lamp providing a white‐coloured light (5000 K, peak of emission around 580 nm). During the test, the light was placed 25 cm over the experimental arena and it provided ~250 lm (bright light), ~62.5 lm (dim light), or it was switched off (darkness). Bright light treatment illuminance was ~3300 lx (values close to those found under tree shade or forest canopy cover in a sunny day; Bhandary et al. 2021), while illuminance in dim light treatments was ~750 lx (similar to values in the typical range of illuminance found within the first metres of cave entrance) (Lunghi et al. 2015; Prous et al. 2004); illuminance values were similar to light intensities eliciting phototactic responses in eyeless cave amphipods (Fišer et al. 2016). The experimental arena consisted in a black and opaque plastic tank (14 × 7.5 × 4.5 cm) filled with 1.5‐cm cave water. A black plastic lid entirely covered half of the arena until the water surface, thus creating a semi‐obscure chamber (dark side), providing a decreasing light gradient from the centre of the arena to the dark side extremity. A coverage box (25 cm × 35 cm) provided with a hole served as lamp physical support allowing light to illuminate the test arena, while preventing light diffuse outside of it (Figure 2). During tests, the experimental arena and its coverage box were placed on a plain surface, kept horizontal by means of a tripod. A total of 27 medium‐size flatworms (12–16 mm) were collected from nearby pools prior to start the experiment and individually placed in 5 × 10 cm plastic tanks filled with 1.5‐cm cave water, where they were let acclimatise for at least 5 min. At the beginning of the test, the focal flatworm was delicately collected by generating a gentle water flow with a pipette to move the animal on a teaspoon. The flatworm was then placed in the centre of the arena, the lid was applied and the covering box placed above it. The lamp was turned on (or not in darkness treatment) and the test started. After 3 min, the coverage box and the arena lid were rapidly removed and the flatworm distance from the centre (0 cm) was registered, with negative values accounting for dark side positions and positive ones for light side positions. This distance was measured by means of a graduated scale on the tank side. Flatworms were then carefully collected and placed back in their respective plastic tanks. During the experiment, each flatworm was exposed to each light‐exposure condition in two replicates, for a total of six tests per individual. Behavioural tests were performed in a randomised order to minimise potential effects related to the sequence of light‐exposure treatments (Altmann 1974; Ferrari et al. 2010; Melotto et al. 2019), while allowing each individual to rest at least 15‐min between consecutive tests. Tests were conducted by three different operators, simultaneously testing the response of a single flatworm. During the experiment, operators were three‐metre apart each other and performed the same light treatment to avoid reciprocal light disturbance and no other light source occurred. Experimental arenas were carefully washed after each test and refilled with cave water to eliminate any trace of previous individuals potentially impacting on flatworm behaviour. Between consecutive tests, researchers operated using headlamps with red light to minimise the disturbance for animals. Test lamp batteries were changed every eight tests to ensure constant illuminance over the experiment. To eliminate the potential confounding effects associated with temperature variations induced by light exposure, we conducted preliminary measurements of water temperature following 3‐min light‐exposure treatments. These tests indicated that the water temperature is unaffected by light exposure and remains stable throughout the 3‐min duration as well as between subsequent treatments. In two cases, flatworm position was recorded with delay and thus these observations were discarded from the analyses (*N* tests analysed = 160). Upon completion of the experiment, all individuals were returned to the pool from which they were originally collected.

### 2.4 Statistical Analyses

We assessed the effect of diurnal cycle on flatworm density using linear mixed models (LMMs). To this extent, we used the number of flatworms detected during the surveys as dependent variable and the phase of the day (day vs. night) as fixed factor. We included the number of operators performing the survey (one or two) as a covariate; as previous analyses evaluated the potential impact of restoration actions on the study population, survey season and intervention actuation (surveys occurred before vs. after it) were included as random effects. In the analyses, the number of active flatworms was square rooted to improve normality.

We used mixed models also to assess flatworm responses to light in experimental tests. Flatworm position (i.e., distance from the centre) was the response variable, and light‐exposure treatment (darkness, dim light, bright light) was included as a fixed factor. Test time (minutes from midnight at the test) was included as an additional covariate to consider diel variation in activity. Individual identity and experimental tank (which also accounted for operator identity) were included in the model as random factors. As we detected a significant effect of light exposure on flatworm position (see Section 3), we used planned orthogonal contrasts (or ‘planned comparisons’) to evaluate the effects of the three exposure treatments. Orthogonal contrasts allow pairwise comparisons between different levels of fixed factors while keeping the probability of type I and type II errors low (Field et al. 2012). In planned contrasts, we first tested whether the darkness treatment differed from the two light treatments (dim and bright light) and then tested differences between dim and bright light conditions.

Statistical analyses were performed in R (version 3.6.0) using *lmerTest*, *lme4* and *glmmTMB* packages (Bates et al. 2015; Brooks et al. 2017; Kuznetsova et al. 2015), while *visreg* package to produce graphs (Breheny et al. 2020).

## 3 Results

### 3.1 Flatworm Abundance and Diurnal Cycle

**FIGURE 3:**
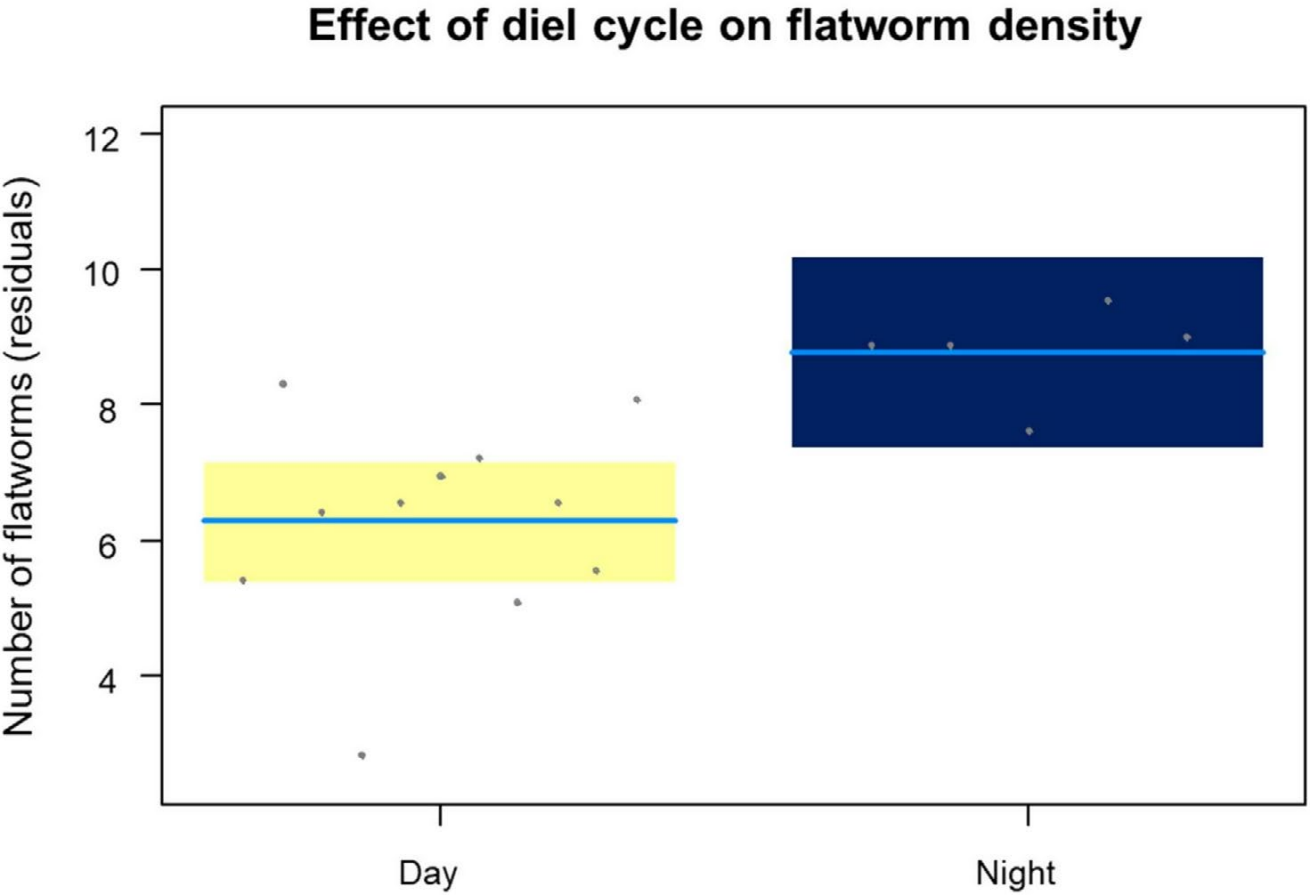
Effect of diurnal cycle on flatworm abundance. Conditional partial residual plots showing the influence of diurnal cycle on the abundance of active *Dendrocoelum italicum* (mixed models). Yellow is for surveys performed during the day phase, whereas blue represents night phase surveys. The number of flatworms used in the model and shown in the graph is square rooted. Shaded areas are 95% confidence bands, solid blue lines represent the estimated mean, while dots are residuals.

The number of detected flatworms ranged from 8 individuals to 109 (mean ± SE: 59.3 ± 14.81: see Table 1). The mean abundance during day and night surveys was 45.5 ± 5.9 and 89.6 ± 9.6 individuals, respectively. Results of mixed models confirmed the significant effect of the diurnal cycle on flatworm abundance (*F*_1,13.0_ = 10.45; *p* = 0.007; Figure 3), with a two‐fold increase in visible flatworms during night counts. The number of operators performing the survey did not significantly affect the number of detected flatworms (*F*_1,13.0_ = 3.65; *p* = 0.078).

### 3.2 Behavioural Response to Light

**FIGURE 4:**
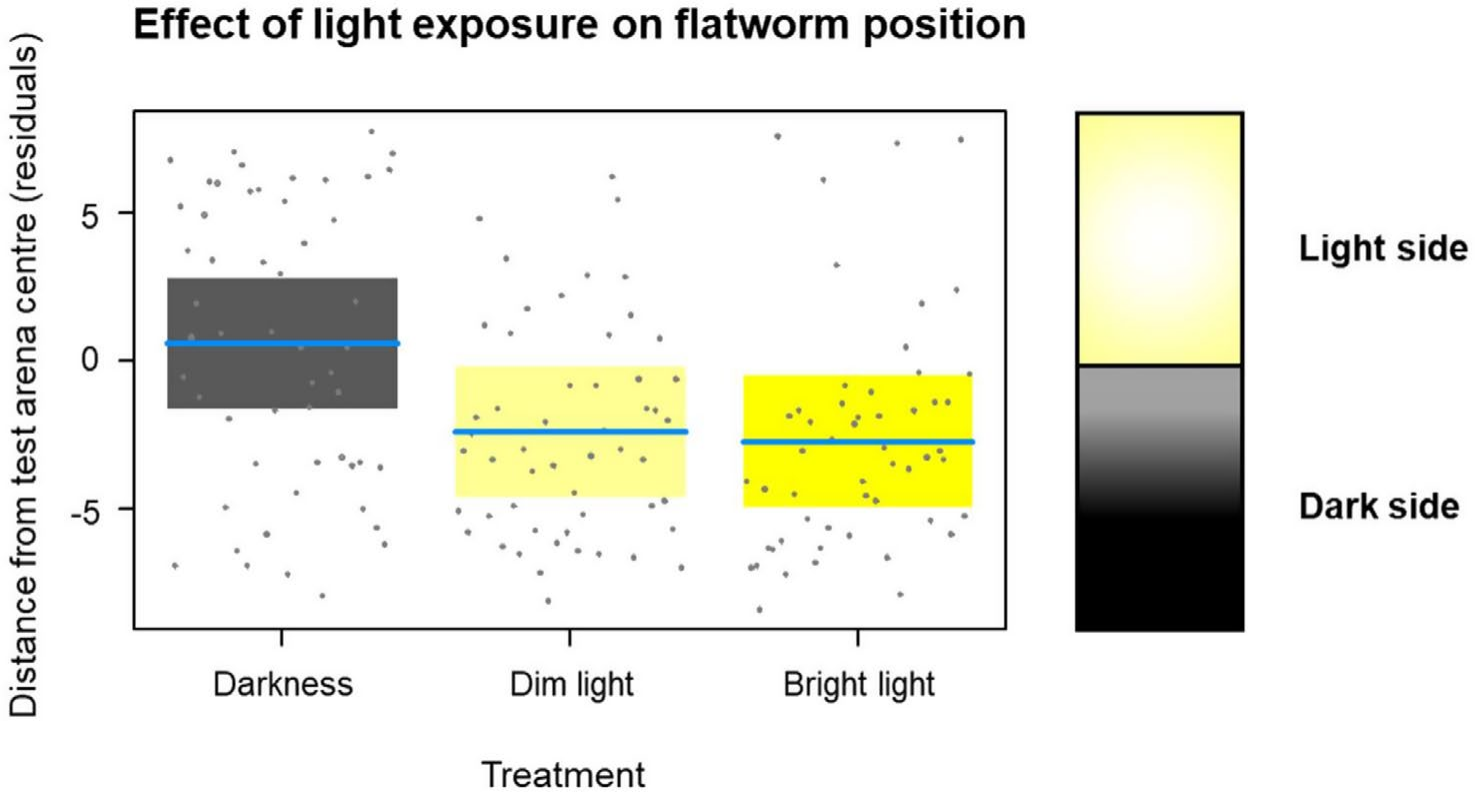
Effect of light‐exposure treatments on flatworm position. Conditional partial residual plots of mixed models showing the influence of light‐exposure treatments on *Dendrocoelum italicum* position within the experimental arena at the end of the test (calculated as distance from the starting central position). Negative values represent preferences for semi‐obscured dark side and positive values are for the illuminated side (see Section 2). Pale yellow is for dim light, dark yellow is bright light while black represent darkness. Shaded areas are 95% confidence bands, solid blue lines represent the estimated mean, whereas dots are residuals. On the right a schematic representation of the experimental arena is aligned with the plot *y* axis.

During behavioural tests, overall flatworm distance from centre of the arena at the end of the trial ranged between the two extreme values (−7 and 7 cm), being on average −1.52 ± 0.12 cm. Mean flatworm position was −2.73 ± 0.55 for bright light and −2.43 ± 0.53 for dim light, while it was not negative (+0.57 ± 0.69) under the darkness treatment. Mixed models (Table 2A) showed that flatworm position was strongly affected by light‐exposure treatments (*F*_2,130.9_ = 10.28; *p* < 0.001, Figure 4). Orthogonal contrasts (Table 2B) revealed that this effect was mainly driven by the very strong differences between light treatments and darkness treatment (*F*_1,130.7_ = 20.42; *p* < 0.001, Figure 4). Conversely, flatworm position was not significantly different between dim light and bright light exposure to (*F*_1,131.2_ = 0.14; *p* = 0.704). Test time did not show significant effects (Table 2A,B).

**TABLE 2:** Influence of light exposure on *Dendrocoelum italicum* position within the experimental arena. (A) Results of linear mixed model including light‐exposure treatments (darkness, dim light, bright light) and daytime (minutes from midnight) as fixed factors. (B) Results of linear mixed model including orthogonal contrasts (see Section 2) to compare light‐exposure treatments on *D. italicum* position (distance from test box centre) at the end of the test.

Independent variable	Estimates	df	*F*	*p*
(A)				
**Light exposure**		**2, 130.9**	**10.28**	**< 0.001**
Daytime	−0.01	1, 24.5	3.98	0.057
(B)				
**Contrast 1**	**−1.05**	**1, 130.7**	**20.42**	**< 0.001**
Contrast 2	−0.15	1, 131.2	0.14	0.704
Daytime	−0.01	1, 24.5	3.98	0.057

*Note:* Contrast 1 is light treatments together (bright + dim light) vs. darkness treatment, while contrast 2 is dim light vs. bright light treatment. For both models, estimates, degrees of freedom, test coefficients and *p* values are reported. Significant effects are in bold.

## 4 Discussion

The results of behavioural tests conducted in this study showed a clear photophobic response of *Dendrocoelum italicum* to light exposure, which seems consistent with the pattern of activity observed under natural conditions. Under both bright and dim light treatments, flatworms moved significantly more towards the darkest part of the experimental arena. These results suggest that flatworms can perceive light even in the absence of any complex visive structure. Light detection can also occur directly at the level of the diencephalon, as demonstrated in different fish species (Breder and Rasquin 1947) and in some troglobiont crustaceans, including *Niphargus* amphipods (Merker 1929). Moreover, light‐sensing extraocular receptors can occur also at the level of the epidermal membrane (Ramirez et al. 2011; Xiang et al. 2010), a fact well reported in both past and recent studies for flatworms (Birkholz and Beane 2017; Rawlinson et al. 2019; Shettigar et al. 2017; Viaud 1948, 1950). For instance, Walter (1907) discovered that surface flatworms in which the eyes had been experimentally removed were still able to perceive light and move away from it, suggesting the existence of extraocular photoreceptors. Since then, several studies provided evidence for the occurrence of photoreceptors at the level of the epidermal membrane in surface triclads, with different species showing a negative phototaxis even when experimentally blinded (Viaud 1948, 1950). More recently, studies focusing on a model animal, the surface flatworm *Schimdtea mediterranea*, provided new insights on structures and mechanisms allowing extraocular light perception in triclads. Rawlinson et al. (2019) showed that opsin (a photopigment through which animals can sense light) is not only located in the eyes of this flatworm species, but that it also occurs around the cerebral ganglia of the adults and near the cilia of the larvae, while Shettigar et al. (2021) identified specialised eye–brain‐independent photoreceptor cells scattered throughout the whole body. Moreover, several studies performed in *S. mediterranea* and other species revealed that different wavelengths or light intensities can trigger specific phototactic behaviours both in eye‐deprived and intact individuals, suggesting the existence of a refined and functional extraocular light‐sensing machinery in surface flatworms (Birkholz and Beane 2017; Davidson et al. 2011; Paskin et al. 2014; Shettigar et al. 2017). However, when concerning cave‐dwelling flatworms, even if a strong sensitivity to light is reported (Vandel 1964), only a few studies assessing their behavioural responses to light exist. Individuals of *Phagocata vitta* , a species that can inhabit groundwaters and has eyes, show marked negative phototaxis even when deprived of the eyes (Viaud 1948). On the contrary, the experiments performed by Buchanan (1936) on *Sphalloplana percaeca*, an eyeless species collected in the Mammoth Cave, did not show any reaction to light stimuli, while individuals quickly died when exposed to sunlight.

Our study indicates that, despite the absence of complex visive structures, *D. italicum* responds to light exposure with a marked photophobic reaction, as individuals showed a strong aversion for the illuminated chamber in both dim and bright light treatments. In line with previous results on surface flatworm species (Davidson et al. 2011), light aversion appeared slightly more pronounced at higher intensity, but differences between dim and bright light treatments were weak and not significant. This lack of differences might depend on the light intensities chosen for this study (dim:bright intensity, 1:4) that may possibly be too close to each other to provide distinct cues to *D. italicum* . Still, the strong photophobic response we observed highlights that even groundwater‐dwelling flatworms with typical troglomorphic adaptations can retain the capability to detect light cues and express light‐mediated behaviours, suggesting the existence of non‐visual photoreceptors also in cave species. Results from preliminary immunochemistry assays seem to suggest that opsin‐like light‐sensitive proteins might be present in *D. italicum* extraocular tissues (i.e., dermal sensing) (A. Melotto et al. unpublished results), but this preliminary observation requires further investigations. Identifying structures and molecular machinery enabling photoreception in *D. italicum* may contribute to shed light on evolutionary processes allowing the subterranean fauna to retain the capability to perceive light stimuli. Photosensitive structures might have adaptive value also in cave organisms as they can allow them to avoid venturing out of the subterranean realm, where conditions encountered can be particularly unsuitable for them (Fišer et al. 2016; Wang et al. 2023). Retention or loss of surface‐related traits in cave fauna can depend on evolutionary trade‐offs balancing energetic expenditure and adaptive advantages associated with their functions. Caves are resource‐depleted environments and loss of non‐functional traits with high metabolic costs should be particularly favoured by natural selection in similar contexts (Moran et al. 2015; Romero 2020; Sumner‐Rooney 2018). In accordance with this prediction, loss of complex visual systems in species exploiting constant darkness regimes is among the most common adaptations. Indeed, development and maintenance of eyes imply elevated energetic costs (Brodrick and Jékely 2023), which can account for up to 5%–15% of the resting metabolism, as shown for some cave fish (Moran et al. 2015). However, if abandoning unnecessarily complex and energetically expensive visual systems is clearly advantageous for troglobiont fauna, relatively simple structures enabling light perception and orientation, such as extraocular photoreceptors, probably have a much lower energetic cost (Brodrick and Jékely 2023) and may be retained, if they can provide some advantages. For instance, Wang et al. (2023) found that both eyed spiders inhabiting cave entrances and eyeless cave‐dwelling congeners exhibit photophobic behaviour and show a conserved functional phototransduction pathway, with little evidence of selection relaxation at the gene expression level. Remarkably, compared to cave‐entrance species, the cave‐dwelling ones showed poor survival when experimentally transplanted in drier cave entrances, suggesting that the capability to respond to light stimuli may retain a key adaptive value for these spiders. Another potential adaptive value of light sensitivity in cave organisms, especially for species living close to the surface, is that it may contribute to modulating the exploitation of ecotones with external environments when conditions are more favourable, as during the night (Mammola and Isaia 2018; Manenti, Galbiati, et al. 2023). In agreement with this hypothesis, our study shows that the activity pattern of *D. italicum* seems to be affected by diel oscillations compatible with circadian rhythm, with a higher number of active individuals during the night (Figure 3). This pattern is observed in many epigean flatworms (Ding et al. 2019; Hinrichsen et al. 2019; Lombardo et al. 2011), which can be sustained even when experimentally decapitated or kept in constant darkness (Itoh and Igarashi 2000; Omond et al. 2017; Omond and Lesku 2023). A marginally non‐significant variation in the activity pattern, with a tendency of occupying dark sectors more frequently during later hours, was also observed during light‐exposure experiments (Table 2A). As for the case of the visual system, maintaining circadian rhythm can be costly for troglobiont species (Moran et al. 2014) and thus is generally expected to be selected against in cave environments; still, several troglobionts maintain circadian rhythm (Beale et al. 2016; de Souza et al. 2024; Friedrich 2013). This is often alleged as some vestigial remaining related to complex pleiotropic effects linked to the preservation of other key metabolic processes and/or presumed to be consistent with patterns of neutral selection. However, exhibiting a circadian rhythm may also have an adaptive value, especially for cave species living close to the interface with surface environments or exploiting small, interconnected crevices that can communicate with them (de Souza et al. 2024; Hoenen and Marques 1998; Tierney et al. 2017), as variation in diel activity can reduce the probability to venture or be drifted out during unfavourable conditions (i.e., during day) (Kureck 1967; Oberrisser and Waringer 2011). This might be the case of *D. italicum* , as it is possible that the diel rhythm shown by flatworms in this study is linked to the proximity to the surface of the microhabitats that they exploit. Indeed, while the main pool seems to remain always in total darkness (0.00 lx), during the day in some nearby puddles a feeble light can occur (maximum 0.04 lx). Moreover, the cave system in the study area is particularly developed and multiple connections with the surface, as well as other interconnected *D. italicum* populations, are likely to exist. We cannot exclude a potential role of diel temperature fluctuations, especially for cave sectors closer to the entrance, or other cues as a possible alternative or complementary zeitgebers mediating the diel activity rhythm observed in *D. italicum* ; still, preliminary analyses including air temperature as an additional covariate did not show a significant effect of temperature on *D. italicum* abundance (*F*_1,7_ = 2.64; *p* = 0.133). Finally, while a marked reaction to visible light in *D. italicum* was evident from behavioural tests, we cannot exclude that other, not visible, wavelengths can play a role in driving the activity patterns observed in the field. Additional studies should investigate how different wavelengths are able to spread across the subterranean environment at different depths, and their possible effect on the activity and space use of cave‐dwelling species.

Studies on cave flatworms offer interesting perspectives for future research on the conservation or loss of circadian rhythmicity. A recent review shows that numerous species of groundwater‐dwelling flatworms, presenting variable degrees of troglomorphism, can also exploit external environments, such as springs (Barzaghi et al. 2021). While for most of them reactions to light or circadian rhythmicity have never been tested, troglomorphic variation might play a relevant role in modulating their activity, space use and patterns of microhabitat selection. For example, despite being riskier due to light exposure and increased predation pressure, springs offer advantages in terms of trophic opportunities compared to resource‐depleted subterranean habitats (Culver and Pipan 2009; Manenti, Forlani, et al. 2023; Manenti, Galbiati, et al. 2023), a factor that can be particularly attractive for mesopredators such as cave‐dwelling flatworms.

Diel rhythmicity and the capability to perceive light are two important but often neglected components regulating the physiology and behaviour of cave‐adapted animals that can come into contact with surface areas or exploit transitioning zones, as this may allow them to avoid suboptimal ecological contexts (Fišer et al. 2016; Friedrich et al. 2011; Wang et al. 2023) or modulate their activity to access alternative environments under favourable conditions (Manenti and Barzaghi 2021). Behavioural experiments comparing species/populations exploiting subterranean habitats and those that occur also in springs or in adjacent surface environments will increase our understanding of mechanisms contributing to maintaining light responsiveness in cave‐dwelling organisms. Tests under natural conditions are an essential tool to investigate behavioural patterns of subterranean species within their ecological contexts; however, generally, they are complicated by logistical challenges posed by the cave environment (Sumner‐Rooney 2018). Our study offers a practical example of an in‐field protocol to assess behavioural responses of groundwater and terrestrial subterranean animals to light. By providing a simple and easily replicable experimental setting that can be used directly in the field, this approach minimises logistical issues while avoiding struggles for setting up experiments in laboratories or mesocosms, which may introduce biases related to transportation or housing, especially for delicate organisms like troglobiont species (Blin et al. 2020). At the same time, even if cave habitats tend to be particularly stable in terms of environmental conditions (Culver and Pipan 2009; Romero 2009), variation in biotic and abiotic features (e.g., resources availability, season, temperature, water condition) can affect troglobiont behaviour and should be considered in field experiments. The experimental setup that we proposed to detect reactions to light stimuli, coupled with quantitative field assessments of the diel activity patterns of subterranean animals, can be a useful approach to investigate variation of these traits and shed light on the process of adaptation to cave life or re‐adaptation to surface life. Moreover, investigating traits, such as photophobia will provide key insights for our understanding of the connections existing between surface and underground realms and possibly contribute to explaining patterns of speciation or species distribution and the existence of small‐scale endemism of some stygobiont species, such as *D. italicum* .

The outcomes of our study call for further investigations to unravel molecular and physiological mechanisms allowing reactions to light stimuli and maintenance of circadian rhythm in cave‐dwelling animals and to experimentally test hypotheses on the evolutionary pathways and trade‐offs linked to surface‐related trait retention.

## Author Contributions

**Benedetta Barzaghi:** conceptualization (equal), investigation (equal), methodology (equal), writing – original draft (supporting), writing – review and editing (equal). **Raoul Manenti:** conceptualization (equal), data curation (equal), investigation (equal), methodology (equal), writing – original draft (supporting), writing – review and editing (equal). **Gentile Francesco Ficetola:** supervision (equal), writing – review and editing (equal). **Roberta Pennati:** supervision (equal), writing – review and editing (equal). **Andrea Melotto:** conceptualization (equal), data curation (equal), formal analysis (equal), investigation (equal), methodology (equal), writing – original draft (lead), writing – review and editing (equal).

## Conflicts of Interest

The authors declare no conflicts of interest.

## Supporting information


Table S1

## Data Availability

Datasets are available in Tables S1 and S2.
