# Supplementary material for: Light in the Darkness: Responses to Light and Diel Activity Rhythm in an Eyeless Cave Flatworm (Dendrocoelum italicum)
Source: Ecol Evol. 2025 Jun 18;15(6):e71584. doi: 10.1002/ece3.71584 (PMC12176500; doi:10.1002/ece3.71584)
Supplement: Supplementary file 1 — Table S1 [file ECE3-15-e71584-s001.docx]

**Supplementary table 1 – Field survey dataset**

Flatworm abundance observed in visual count surveys in the “Bus del Budrio” cave. Reported in the table: survey identity, date of survey, season, phase of the day in which the survey was conducted (night or day), number of observed flatworms during the visual count, number of operators involved in the visual count, water condition (visibility during counts), survey occurrence with respect to the restoration intervention performed (see Manenti et al. 2019), and observation exclusion from the analyses (due to water condition hampering flatworm count). Barred surveys were not included in the analyses as turbid water conditions hampered reliable flatworm counts.

| **Survey_id** | **Date** | **Season** | **Day_phase** | **N_flatworms** | **N_operators** | **Water_ condition** | **Intervention** | **Exclusion** |
| --- | --- | --- | --- | --- | --- | --- | --- | --- |
| 1 | 04/02/2016 | winter | day | 49 | 1 | clear | before | no |
| 2 | 22/04/2016 | spring | day | 69 | 2 | clear | before | no |
| 3 | 27/07/2016 | summer | night | 109 | 1 | clear | before | no |
| 4 | 15/11/2016 | autumn | day | 41 | 2 | clear | before | no |
| 5 | 18/11/2016 | autumn | day | 8 | 2 | clear | before | no |
| 6 | 27/11/2016 | autumn | day | 43 | 2 | clear | before | no |
| 7 | 03/12/2016 | autumn | day | 73 | 1 | clear | before | no |
| 8 | 04/12/2016 | autumn | day | 6 | 2 | turbid | after | yes |
| 9 | 25/01/2017 | winter | day | 7 | 2 | turbid | after | yes |
| 10 | 19/03/2017 | spring | day | 52 | 2 | clear | after | no |
| 11 | 22/03/2017 | spring | night | 109 | 1 | clear | after | no |
| 12 | 30/03/2017 | spring | day | 26 | 2 | clear | after | no |
| 13 | 30/03/2017 | spring | night | 58 | 2 | clear | after | no |
| 14 | 31/03/2017 | spring | day | 43 | 2 | clear | after | no |
| 15 | 31/03/2017 | spring | night | 91 | 2 | clear | after | no |
| 16 | 01/04/2017 | winter | day | 31 | 2 | clear | after | no |
| 17 | 24/01/2018 | winter | day | 65 | 2 | clear | after | no |
| 18 | 24/01/2018 | winter | night | 81 | 2 | clear | after | no |

**Bibliography**

Manenti, R., Barzaghi, B., Tonni, G., Ficetola, G. F., & Melotto, A. (2019). Even worms matter: Cave habitat restoration for a planarian species increased environmental suitability but not abundance. ORYX, 53(2), 216-221. doi:10.1017/S0030605318000741

**Supplementary table 2 – Behavioural test dataset**

Flatworm position in experimental arena during light exposure tests. Reported in the table: Round of the behavioural test (three test were conducted in parallel by three different operators during each round), light exposure treatment, time at the test, time as minutes from midnight, test replicate (each individual was exposed to each treatment twice), flatworm position from the centre of the experimental arena (cm), flatworm identity, identity of the experimental arena and the operator performing the test.

| **Test_round** | **Treatment** | **Time** | **Minutes_from_midnight** | **Replicate** | **Position(cm)** | **Flatworm_id** | **Arena_operator** |
| --- | --- | --- | --- | --- | --- | --- | --- |
| 1 | Darkness | 15:11 | 911 | 1 | 7 | P1 | 1 |
| 2 | Bright light | 15:19 | 919 | 1 | -4 | P2 | 1 |
| 3 | Dim light | 15:28 | 928 | 1 | -5 | P3 | 1 |
| 4 | Dim light | 15:36 | 936 | 1 | -3 | P1 | 1 |
| 5 | Darkness | 15:41 | 941 | 1 | -7 | P2 | 1 |
| 6 | Bright light | 15:48 | 948 | 1 | -7 | P3 | 1 |
| 7 | Bright light | 15:57 | 957 | 1 | -7 | P1 | 1 |
| 8 | Dim light | 16:05 | 965 | 1 | -6 | P2 | 1 |
| 9 | Darkness | 16:12 | 972 | 1 | 5 | P3 | 1 |
| 10 | Darkness | 16:18 | 978 | 2 | 1 | P1 | 1 |
| 11 | Bright light | 16:24 | 984 | 2 | -7 | P2 | 1 |
| 12 | Dim light | 16:29 | 989 | 2 | -1 | P3 | 1 |
| 13 | Dim light | 16:37 | 997 | 2 | -0.5 | P1 | 1 |
| 14 | Darkness | 16:43 | 1003 | 2 | 5 | P2 | 1 |
| 15 | Bright light | 16:48 | 1008 | 2 | -3 | P3 | 1 |
| 16 | Bright light | 16:54 | 1014 | 2 | -5 | P1 | 1 |
| 17 | Dim light | 17:00 | 1020 | 2 | 6 | P2 | 1 |
| 18 | Darkness | 17:06 | 1026 | 2 | 2 | P3 | 1 |
| 19 | Bright light | 17:38 | 1058 | 1 | -7 | P10 | 1 |
| 20 | Dim light | 17:45 | 1065 | 1 | 0.5 | P11 | 1 |
| 21 | Darkness | 17:50 | 1070 | 1 | 1.5 | P12 | 1 |
| 22 | Dim light | 17:57 | 1077 | 1 | -6 | P10 | 1 |
| 23 | Darkness | 18:02 | 1082 | 1 | -2 | P11 | 1 |
| 24 | Bright light | 18:09 | 1089 | 1 | 7 | P12 | 1 |
| 25 | Darkness | 18:16 | 1096 | 1 | 4 | P10 | 1 |
| 26 | Bright light | 18:22 | 1102 | 1 | -7 | P11 | 1 |
| 27 | Dim light | 18:28 | 1108 | 1 | -4 | P12 | 1 |
| 28 | Dim light | 18:34 | 1114 | 2 | NA | P10 | 1 |
| 29 | Bright light | 18:40 | 1120 | 2 | -6.5 | P11 | 1 |
| 30 | Darkness | 18:46 | 1126 | 2 | 7 | P12 | 1 |
| 31 | Darkness | 18:54 | 1134 | 2 | 4 | P10 | 1 |
| 32 | Dim light | 18:59 | 1139 | 2 | -1 | P11 | 1 |
| 33 | Bright light | 19:05 | 1145 | 2 | -1 | P12 | 1 |
| 34 | Bright light | 19:11 | 1151 | 2 | -4 | P10 | 1 |
| 35 | Darkness | 19:17 | 1157 | 2 | 6.5 | P11 | 1 |
| 36 | Dim light | 19:23 | 1163 | 2 | -5.5 | P12 | 1 |
| 37 | Bright light | 19:55 | 1195 | 1 | -3 | P19 | 1 |
| 38 | Darkness | 20:01 | 1201 | 1 | -1 | P20 | 1 |
| 39 | Dim light | 20:07 | 1207 | 1 | 2 | P21 | 1 |
| 40 | Dim light | 20:13 | 1213 | 1 | -0.5 | P19 | 1 |
| 41 | Bright light | 20:18 | 1218 | 1 | -5 | P20 | 1 |
| 42 | Darkness | 20:24 | 1224 | 1 | -6.5 | P21 | 1 |
| 43 | Darkness | 20:30 | 1230 | 1 | -3.5 | P19 | 1 |
| 44 | Dim light | 20:36 | 1236 | 1 | -7 | P20 | 1 |
| 45 | Bright light | 20:42 | 1242 | 1 | -7 | P21 | 1 |
| 46 | Bright light | 20:47 | 1247 | 2 | -2 | P19 | 1 |
| 47 | Dim light | 20:52 | 1252 | 2 | -7 | P20 | 1 |
| 48 | Darkness | 20:59 | 1259 | 2 | 7 | P21 | 1 |
| 49 | Dim light | 21:05 | 1265 | 2 | -3 | P19 | 1 |
| 50 | Darkness | 21:10 | 1270 | 2 | -7 | P20 | 1 |
| 51 | Bright light | 21:17 | 1277 | 2 | -7 | P21 | 1 |
| 52 | Darkness | 21:22 | 1282 | 2 | 6.5 | P19 | 1 |
| 53 | Bright light | 21:27 | 1287 | 2 | -7 | P20 | 1 |
| 54 | Dim light | 21:33 | 1293 | 2 | 1.5 | P21 | 1 |
| 1 | Darkness | 15:11 | 911 | 1 | -7 | P4 | 2 |
| 2 | Bright light | 15:19 | 919 | 1 | 6.5 | P5 | 2 |
| 3 | Dim light | 15:28 | 928 | 1 | -4 | P6 | 2 |
| 4 | Dim light | 15:36 | 936 | 1 | -6 | P4 | 2 |
| 5 | Darkness | 15:41 | 941 | 1 | 6 | P5 | 2 |
| 6 | Bright light | 15:48 | 948 | 1 | -6 | P6 | 2 |
| 7 | Bright light | 15:57 | 957 | 1 | -3 | P4 | 2 |
| 8 | Dim light | 16:05 | 965 | 1 | -7 | P5 | 2 |
| 9 | Darkness | 16:12 | 972 | 1 | -4 | P6 | 2 |
| 10 | Darkness | 16:18 | 978 | 2 | 7 | P4 | 2 |
| 11 | Bright light | 16:24 | 984 | 2 | 5 | P5 | 2 |
| 12 | Dim light | 16:29 | 989 | 2 | -1 | P6 | 2 |
| 13 | Dim light | 16:37 | 997 | 2 | -7 | P4 | 2 |
| 14 | Darkness | 16:43 | 1003 | 2 | 5 | P5 | 2 |
| 15 | Bright light | 16:48 | 1008 | 2 | -0.5 | P6 | 2 |
| 16 | Bright light | 16:54 | 1014 | 2 | 0.2 | P4 | 2 |
| 17 | Dim light | 17:00 | 1020 | 2 | -2 | P5 | 2 |
| 18 | Darkness | 17:06 | 1026 | 2 | -5 | P6 | 2 |
| 19 | Bright light | 17:38 | 1058 | 1 | -7 | P13 | 2 |
| 20 | Dim light | 17:45 | 1065 | 1 | -7 | P14 | 2 |
| 21 | Dim light | 17:50 | 1070 | 1 | 1.5 | P15 | 2 |
| 22 | Dim light | 17:57 | 1077 | 1 | -7 | P13 | 2 |
| 23 | Darkness | 18:02 | 1082 | 1 | 0 | P14 | 2 |
| 24 | Bright light | 18:09 | 1089 | 1 | -3 | P15 | 2 |
| 25 | Darkness | 18:16 | 1096 | 1 | -3 | P13 | 2 |
| 26 | Bright light | 18:22 | 1102 | 1 | -3 | P14 | 2 |
| 27 | Darkness | 18:28 | 1108 | 1 | 2 | P15 | 2 |
| 28 | Dim light | 18:34 | 1114 | 2 | -0.5 | P13 | 2 |
| 29 | Bright light | 18:40 | 1120 | 2 | -3.5 | P14 | 2 |
| 30 | Darkness | 18:46 | 1126 | 2 | 6 | P15 | 2 |
| 31 | Darkness | 18:54 | 1134 | 2 | -7 | P13 | 2 |
| 32 | Dim light | 18:59 | 1139 | 2 | -4 | P14 | 2 |
| 33 | Bright light | 19:05 | 1145 | 2 | -4 | P15 | 2 |
| 34 | Bright light | 19:11 | 1151 | 2 | -1 | P13 | 2 |
| 35 | Darkness | 19:17 | 1157 | 2 | 6.5 | P14 | 2 |
| 36 | Dim light | 19:23 | 1163 | 2 | -6 | P15 | 2 |
| 37 | Bright light | 19:55 | 1195 | 1 | -6.5 | P22 | 2 |
| 38 | Darkness | 20:01 | 1201 | 1 | -6 | P23 | 2 |
| 39 | Dim light | 20:07 | 1207 | 1 | -7 | P24 | 2 |
| 40 | Dim light | 20:13 | 1213 | 1 | 1 | P22 | 2 |
| 41 | Bright light | 20:18 | 1218 | 1 | -3.5 | P23 | 2 |
| 42 | Darkness | 20:24 | 1224 | 1 | -1.5 | P24 | 2 |
| 43 | Darkness | 20:30 | 1230 | 1 | 2 | P22 | 2 |
| 44 | Dim light | 20:36 | 1236 | 1 | -5 | P23 | 2 |
| 45 | Bright light | 20:42 | 1242 | 1 | -5 | P24 | 2 |
| 46 | Bright light | 20:47 | 1247 | 2 | -7 | P22 | 2 |
| 47 | Dim light | 20:52 | 1252 | 2 | -1 | P23 | 2 |
| 48 | Darkness | 20:59 | 1259 | 2 | -2 | P24 | 2 |
| 49 | Dim light | 21:05 | 1265 | 2 | -7 | P22 | 2 |
| 50 | Darkness | 21:10 | 1270 | 2 | -1 | P23 | 2 |
| 51 | Bright light | 21:17 | 1277 | 2 | -4 | P24 | 2 |
| 52 | Darkness | 21:22 | 1282 | 2 | -4 | P22 | 2 |
| 53 | Bright light | 21:27 | 1287 | 2 | 7 | P23 | 2 |
| 54 | Dim light | 21:33 | 1293 | 2 | -3 | P24 | 2 |
| 1 | Darkness | 15:11 | 911 | 1 | -7 | P7 | 3 |
| 2 | Bright light | 15:19 | 919 | 1 | -7 | P8 | 3 |
| 3 | Dim light | 15:28 | 928 | 1 | 1.5 | P9 | 3 |
| 4 | Dim light | 15:36 | 936 | 1 | 7 | P7 | 3 |
| 5 | Darkness | 15:41 | 941 | 1 | 6.8 | P8 | 3 |
| 6 | Bright light | 15:48 | 948 | 1 | 1 | P9 | 3 |
| 7 | Bright light | 15:57 | 957 | 1 | -3 | P7 | 3 |
| 8 | Dim light | 16:05 | 965 | 1 | 6 | P8 | 3 |
| 9 | Darkness | 16:12 | 972 | 1 | 0 | P9 | 3 |
| 10 | Darkness | 16:18 | 978 | 2 | 7 | P7 | 3 |
| 11 | Bright light | 16:24 | 984 | 2 | 0.5 | P8 | 3 |
| 12 | Dim light | 16:29 | 989 | 2 | -1 | P9 | 3 |
| 13 | Dim light | 16:37 | 997 | 2 | 5 | P7 | 3 |
| 14 | Darkness | 16:43 | 1003 | 2 | 1 | P8 | 3 |
| 15 | Bright light | 16:48 | 1008 | 2 | 1.5 | P9 | 3 |
| 16 | Bright light | 16:54 | 1014 | 2 | 4 | P7 | 3 |
| 17 | Dim light | 17:00 | 1020 | 2 | 3.5 | P8 | 3 |
| 18 | Darkness | 17:06 | 1026 | 2 | -1.5 | P9 | 3 |
| 19 | Bright light | 17:38 | 1058 | 1 | -3.5 | P16 | 3 |
| 20 | Dim light | 17:45 | 1065 | 1 | -7 | P17 | 3 |
| 21 | Darkness | 17:50 | 1070 | 1 | 0 | P18 | 3 |
| 22 | Dim light | 17:57 | 1077 | 1 | -1 | P16 | 3 |
| 23 | Darkness | 18:02 | 1082 | 1 | -4 | P17 | 3 |
| 24 | Bright light | 18:09 | 1089 | 1 | -6 | P18 | 3 |
| 25 | Darkness | 18:16 | 1096 | 1 | 1.5 | P16 | 3 |
| 26 | Bright light | 18:22 | 1102 | 1 | -2 | P17 | 3 |
| 27 | Dim light | 18:28 | 1108 | 1 | -4 | P18 | 3 |
| 28 | Dim light | 18:34 | 1114 | 2 | -0.5 | P16 | 3 |
| 29 | Bright light | 18:40 | 1120 | 2 | -2 | P17 | 3 |
| 30 | Darkness | 18:46 | 1126 | 2 | -2.5 | P18 | 3 |
| 31 | Darkness | 18:54 | 1134 | 2 | -4 | P16 | 3 |
| 32 | Dim light | 18:59 | 1139 | 2 | -4 | P17 | 3 |
| 33 | Bright light | 19:05 | 1145 | 2 | -2.5 | P18 | 3 |
| 34 | Bright light | 19:11 | 1151 | 2 | -0.5 | P16 | 3 |
| 35 | Darkness | 19:17 | 1157 | 2 | 7 | P17 | 3 |
| 36 | Dim light | 19:23 | 1163 | 2 | -1 | P18 | 3 |
| 37 | Bright light | 19:55 | 1195 | 1 | -7 | P25 | 3 |
| 38 | Darkness | 20:01 | 1201 | 1 | 7 | P26 | 3 |
| 39 | Dim light | 20:07 | 1207 | 1 | -0.5 | P27 | 3 |
| 40 | Dim light | 20:13 | 1213 | 1 | -6 | P25 | 3 |
| 41 | Bright light | 20:18 | 1218 | 1 | 1.5 | P26 | 3 |
| 42 | Darkness | 20:24 | 1224 | 1 | -7 | P27 | 3 |
| 43 | Darkness | 20:30 | 1230 | 1 | -5 | P25 | 3 |
| 44 | Dim light | 20:36 | 1236 | 1 | -3 | P26 | 3 |
| 45 | Bright light | 20:42 | 1242 | 1 | 6 | P27 | 3 |
| 46 | Bright light | 20:47 | 1247 | 2 | -5 | P25 | 3 |
| 47 | Dim light | 20:52 | 1252 | 2 | -5 | P26 | 3 |
| 48 | Darkness | 20:59 | 1259 | 2 | -6 | P27 | 3 |
| 49 | Dim light | 21:05 | 1265 | 2 | -0.5 | P25 | 3 |
| 50 | Darkness | 21:10 | 1270 | 2 | 7 | P26 | 3 |
| 51 | Bright light | 21:17 | 1277 | 2 | NA | P27 | 3 |
| 52 | Darkness | 21:22 | 1282 | 2 | 7 | P25 | 3 |
| 53 | Bright light | 21:27 | 1287 | 2 | 0 | P26 | 3 |
| 54 | Dim light | 21:33 | 1293 | 2 | -7 | P27 | 3 |
